# ADAM-17 Activity and Its Relation to ACE2: Implications for Severe COVID-19

**DOI:** 10.3390/ijms25115911

**Published:** 2024-05-29

**Authors:** Jiangming Sun, Andreas Edsfeldt, Joel Svensson, Toralph Ruge, Isabel Goncalves, Per Swärd

**Affiliations:** 1 Cardiovascular Research-Translational Studies, Department of Clinical Sciences Malmö, Lund University, 205 02 Malmö, Sweden; jiangming.sun@med.lu.se (J.S.); andreas.edsfeldt@med.lu.se (A.E.); isabel.goncalves@med.lu.se (I.G.); 2 Department of Cardiology, Skåne University Hospital, 205 02 Malmö, Sweden; 3 Wallenberg Center for Molecular Medicine, Lund University, 221 00 Lund, Sweden; 4 Department of Laboratory Medicine, Lund University, 221 00 Lund, Sweden; joel.svensson@med.lu.se; 5 Department of Emergency and Internal Medicine, Skånes University Hospital, 214 28 Malmö, Sweden; toralph.ruge@med.lu.se; 6 Department of Clinical Sciences Malmö, Lund University, 214 28 Malmö, Sweden; 7 Department of Internal Medicine, Skåne University Hospital, 214 28 Malmö, Sweden; 8 Clinical and Molecular Osteoporosis Research Unit, Departments of Orthopedics and Clinical Sciences, Skåne University Hospital, Lund University, 205 02 Malmö, Sweden

**Keywords:** angiotensin-converting enzyme 2, a disintegrin and metalloproteinase-17, coronavirus disease 2019, severe acute respiratory syndrome coronavirus 2

## Abstract

There is a lack of studies aiming to assess cellular a disintegrin and metalloproteinase-17 (ADAM-17) activity in COVID-19 patients and the eventual associations with the shedding of membrane-bound angiotensin-converting enzyme 2 (mACE2). In addition, studies that investigate the relationship between ACE2 and ADAM-17 gene expressions in organs infected by SARS-CoV-2 are lacking. We used data from the Massachusetts general hospital COVID-19 study (306 COVID-19 patients and 78 symptomatic controls) to investigate the association between plasma levels of 33 different ADAM-17 substrates and COVID-19 severity and mortality. As a surrogate of cellular ADAM-17 activity, an ADAM-17 substrate score was calculated. The associations between soluble ACE2 (sACE2) and the ADAM-17 substrate score, renin, key inflammatory markers, and lung injury markers were investigated. Furthermore, we used data from the Genotype-Tissue Expression (GTEx) database to evaluate *ADAM-17* and *ACE2* gene expressions by age and sex in ages between 20–80 years. We found that increased ADAM-17 activity, as estimated by the ADAM-17 substrates score, was associated with COVID-19 severity (*p* = 0.001). ADAM-17 activity was also associated with increased mortality but did not reach statistical significance (*p* = 0.06). Soluble ACE2 showed the strongest positive correlation with the ADAM-17 substrate score, follow by renin, interleukin-6, and lung injury biomarkers. The ratio of *ADAM-17* to *ACE2* gene expression was highest in the lung. This study indicates that increased ADAM-17 activity is associated with severe COVID-19. Our findings also indicate that there may a bidirectional relationship between membrane-bound ACE2 shedding via increased ADAM-17 activity, dysregulated renin–angiotensin system (RAS) and immune signaling. Additionally, differences in *ACE2* and *ADAM-17* gene expressions between different tissues may be of importance in explaining why the lung is the organ most severely affected by COVID-19, but this requires further evaluation in prospective studies.

## 1. Introduction

Infection with severe acute respiratory syndrome coronavirus 2 (SARS-CoV-2) can range from asymptomatic to severe pneumonia and acute respiratory distress syndrome (ARDS) [1], where ARDS is particularly associated with high mortality [2,3]. There are several extrapulmonary manifestations associated with disease severity and mortality from COVID-19, including acute kidney injury, cardiac and thromboembolic complications [1]. Angiotensin-converting enzyme 2 (ACE2), the target receptor for SARS-CoV-2, is expressed by several airway epithelial cell types, including type I and type II alveolar cells in the lungs, thereby providing a rationale for why the virus has the affinity to affect the lungs [4,5]. However, ACE2 is also expressed in other cell types [1,4,5], partly explaining why SARS-CoV-2 also may induce organ-specific pathology in the heart [6], the kidneys [7], the vascular system (with increased risk of both arterial and venous thrombosis) [8,9,10], as well as the intestines [11]. Thus, direct viral toxicity may be associated with multi-organ injury [1].

The risk of severe COVID-19 and associated mortality seems to increase exponentially with increasing age and is more frequent in men than in women [2,3,12]. The highest risk of severe COVID-19 and associated mortality is observed in men >70 years, patients with obesity, hypertension, cardiovascular disease and/or diabetes [3,12,13]. The underlying pathophysiology behind these clinical observations is not known, but potentially important for improving treatments.

The entrance of SARS-CoV-2 in the host cell is possible through attachment of the S1 region of the S-protein to the active surface domain of membrane-bound ACE2 (mACE2) [14], after which the S2 region of the virus S protein enables fusion of the virus and the host cell membrane [14]. Infection leads to increased a disintegrin and metalloproteinase-17 (ADAM-17) activity, which can induce the shedding of mACE2 and induce pro-inflammatory pathways, by the shedding of several membrane-bound proteins such as tumor necrosis factor (TNF), interleukin 6 receptor (IL6R) and TNF receptors [15].

There are indications that the interaction between ADAM-17 activity and the SARS-CoV-2 receptor ACE2 plays a crucial part in the progression to severe COVID-19 [16,17,18,19]. Increased ADAM-17 activity is implicated in the progression of multiple chronic diseases [20], diseases that are also associated with an increased risk of severe COVID-19 [3,12,13]. However, most of these studies are based on genetic associations, or on ADAM-17 inhibition in mouse or in vitro [17,20,21]. In humans, findings indicate that high levels of ADAM-17 substrates (including ACE2) are associated with severe COVID-19 [16,22,23]. In addition, an increased genetic susceptibility to elevated levels of plasma ADAM-17 (the extracellular domain) is associated with a higher risk of severe COVID-19 [18]. Linking high ADAM-17 activity to the risk of severe COVID-19 in humans would strengthen the potential role of ADAM-17 inhibition as a therapeutic target in COVID-19.

Mechanisms underlying increased levels of sACE2 in severe COVID-19 are unclear but could be related to increased ADAM-17 activity [16,24], hyperinflammation (elevated levels of plasma IL-1beta, IL-6 and TNF-alpha) [25], increased renin–angiotensin system activity [26], and lung cell injury [27].

The aim of this study was to investigate if ADAM-17 substrates in plasma are increased in severe COVID-19 and to what extent they are correlated with sACE2. We also explored if there are concurrent age- and sex-related gene expression changes in target organs. Additionally, we examined the correlations between sACE2, key inflammatory and lung injury markers, and renin. Data from the openly accessible Massachusetts general hospital (MGH) COVID-19 study [28], and the GTEx database were used for these analyses. We hypothesized that ADAM-17 substrates in plasma are increased in severe COVID-19, suggestive of increased cellular ADAM-17 activity, and strongly correlated with sACE2.

## 2. Results

### 2.1. Indications of Increased ADAM-17 Activity in Patients with Severe COVID-19

**Figure 1:**
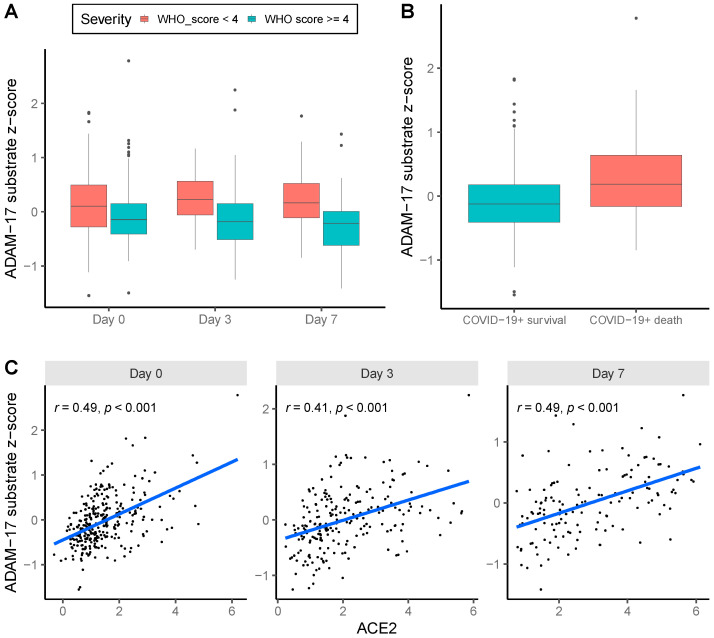
The estimated ADAM-17 activity in the relationship with COVID-19: (**A**) ADAM-17 substrate z-scores are higher in COVID-19 patients with severe disease on days 0 (n = 305, of whom n = 80 had severe COVID-19), 3 (n = 214, of whom n = 75 had severe COVID-19) and 7 (n = 138, of whom n = 68 had severe COVID-19). For the analyses of the present study, we defined patients with scores 1–3 as with severe illness, and scores 4–6 as non-severe illness, based on the WHO COVID-19 outcome scale; and (**B**) ADAM-17 substrate z-scores at baseline (day 0) are higher in patients who died from COVID-19 (n = 42) than COVID-19+ survivals (n = 263) until day 28. (**C**) ADAM-17 substrate z-scores positively correlate with soluble ACE2 on days 0, 3 and 7, respectively.

Based on the MGH-OLINK-COVID-19 dataset, the ADAM-17 substrate z-score was overall (day 0, day 3 and day 7 altogether) higher in patients with severe COVID-19 (OR = 1.45 per SD 95% CI 1.09–1.93, *p* = 0.01, Figure 1A) adjusting for comorbidities, age, and BMI categories. Furthermore, the ADAM-17 z-score at baseline predicted mortality within 28 days adjusted by age categories and BMI categories (OR = 1.47, 95% CI 1.01–2.13, *p* = 0.041, Figure 1B) but did not remain significant upon adjusting for comorbidities (OR = 1.47 per SD 95% CI 0.98–2.22, *p* = 0.064). No significant difference was found for the ADAM-17 substrate z-scores in COVID-19 patients compared to controls at baseline (OR = 0.82, 95% CI 0.50–1.34, *p* = 0.43). The ADAM-17 substrate z-score was positively correlated with sACE2 (R2 = 0.215, 95% CI 0.167–0.266, *p* = 1.6 × 10^−30^, Figure 1C).

**Figure 2:**
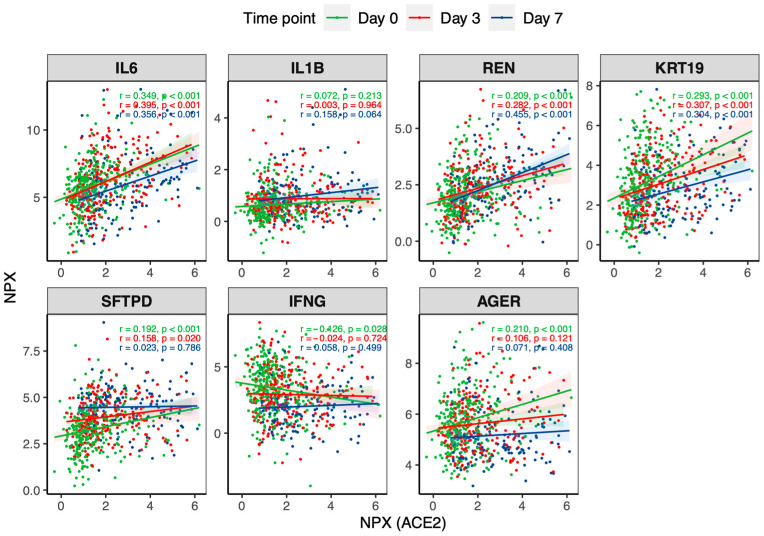
Correlation between ACE2 and protein levels of TNF, IL6, IL1B, REN, KRT19, SFTPD, IFNG and AGER at days 0 (n = 383), 3 (n = 218) and 7 (n = 138). NPX: normalized protein expression.

After adjusting for comorbidities, age and BMI categories, the overall correlation (day 0, day 3 and day 7 altogether) between sACE2 and renin (R2 = 0.134 95% CI 0.092–0.180, *p* = 1.9 × 10^−19^), IL-6 (R2 = 0.071 95% CI 0.040–0.109, *p* = 1.6 × 10^−12^), KRT19 (R2 = 0.035 95% CI 0.014–0.065, *p* = 4.2 × 10^−6^) and SP-D (R2 = 0.019 95% CI 0.005–0.043, *p* = 7.8 × 10^−4^) was positive. (Figure 2). No statistically significant correlation (day 0, day 3, day 7 altogether) between plasma ACE2 levels and IFN gamma (R2 = 0.00002 95% CI 0.0–0.008, *p* = 0.72) and AGER (R2 = 0.002 95% CI 0.00–0.014, *p* = 0.23) was found. However, ACE2 was correlated with IFN-gamma (R2 = 0.011 95% CI 0.000–0.041, *p* = 0.044) and AGER (R2 = 0.024 95% CI 0.003–0.063, *p* = 2.7 × 10^−3^) on admission (day 0) but not on days 3 or 7.

### 2.2. Differences in Gene Expression of ACE2 and ADAM-17 between Different Tissues

**Figure 3:**
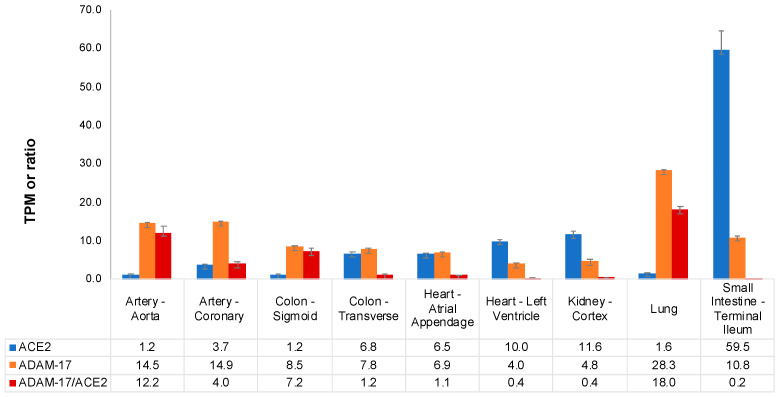
Expression of *ACE2*, *ADAM-17* and the ratio in various human tissues. Gene expression/ratio is displayed as mean +/− SEM. Artery—Aorta (N = 432), Artery—Coronary (N = 240), Colon—Sigmoid (N = 373), Colon—Transverse (N = 406), Heart—Atrial Appendage (N = 429), Heart—Left Ventricle (N = 432), Kidney—Cortex (N = 85), Lung (N = 578), Small Intestine—Terminal Ileum (N = 187). TPM: transcripts per million; *ACE2*: angiotensin-converting enzyme 2; *ADAM-17*: a disintegrin and metalloproteinase 17.

We investigated GTEx human tissues where there are implications of tissue-specific involvement in COVID-19, namely, the lungs (N = 515), the arteries (aorta (N = 387) and coronary arteries (N = 213)), the heart (atrial appendage (N = 372) and left ventricle (N = 382)), the kidney (cortex, N = 73), the colon (transverse (N = 368) and sigmoid (N = 318)), and small intestine (N = 174). Based on the gene expression levels from GTEx, *ACE2* gene expression was highest in the small intestine, followed by the kidneys and the cardiac left ventricle. Moderate gene expression was found in the atrial appendage of the heart and the transverse colon, whereas low gene expression was found in the lungs and the arteries. Of the investigated tissues, the *ADAM-17* gene expression was highest in the lung (Figure 3).

In the tissues investigated in the present study, the ratio between *ADAM-17* and *ACE2* gene expression was highest in the lung (18.0 times) followed by the aorta (12.2 times), sigmoid colon (7.2 times), coronary artery (4.0 times), transverse colon (1.2 times), atrial appendage (1.1 times), left ventricle (0.4 times), kidney (0.4 times) and small intestine (0.2 times) (Figure 3).

### 2.3. Age and Sex Differences in the ACE2 and ADAM-17 Gene Expression

**Figure 4:**
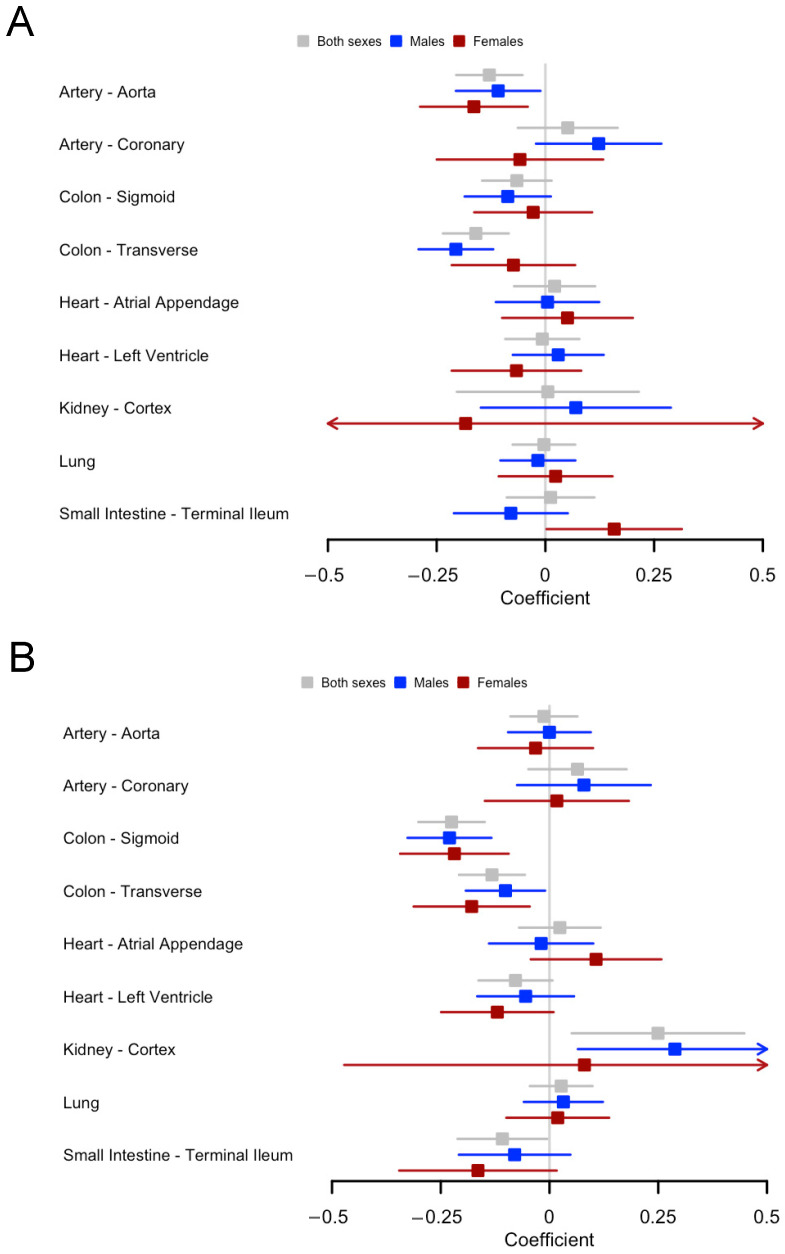
Gene expression of (**A**) *ACE2*; and (**B**) *ADAM-17* varies by age of donors in various human tissues. The coefficients with 95% confidence intervals were displayed. The coefficients were obtained by regressing gene expression on age group of donors adjusted for sequencing platform, sequencing protocol and sex, if both males and females were considered. Details are given in the Supplementary Materials Tables S1 and S3.

For *ACE2*, increasing age group was associated with lower *ACE2* gene expression in the aorta (β = −0.13, *p* = 0.001) and the transverse colon (β = −0.16, *p* = 4.2 × 10^−5^) for both sexes (Figure 4A, Supplementary Materials Table S1). A significant interaction effect for age group and sex was found in the terminal ileum (*p* = 0.02); i.e., the ACE2 expression increased more with increasing age in females than in males. Other than that, there were no significant interactions between age and sex in any of the investigated tissues (Supplementary Materials Table S2).

Regarding *ADAM-17*, increasing age group was associated with higher *ADAM-17* gene expression in kidney tissue (β = 0.25, *p* = 0.016) for both sexes, and lower *ADAM-17* gene expression in the sigmoid (β = −0.23, *p* = 1.8 × 10^−8^) and transverse (β = −0.13, *p* = 7 × 10^−4^) colon, and the terminal ileum (β = −0.11, *p* = 0.04) for both sexes. (Figure 4B, Supplementary Materials Table S3). There were no sex-related differences in age-varied ADAM-17 gene expression in any of the tissues of interest (Supplementary Materials Table S4).

## 3. Discussion

The present study suggests that increased ADAM-17 activity, as estimated by the ADAM-17 substrates score, is associated with increased severity of COVID-19. ADAM-17 activity was also associated with increased mortality, although this did not reach statistical significance. Several chronic diseases, such as chronic inflammatory and cardiovascular disease, are associated with increased ADAM-17 activity [20]. For example, genetic associations have been observed between elevated plasma levels of ADAM-17 and rheumatoid arthritis [18]. Furthermore, although a genetic predisposition to elevated circulating ADAM-17 levels is associated with severe COVID-19 [18], and ADAM-17 inhibition in mice has been shown to offer a protective role against morbidity, lung injury, and inflammation upon SARS-CoV-2 infection [17], further human studies are needed. The findings of the present study contribute to the existing knowledge by indicating that the selective inhibition of ADAM-17 could have potential therapeutic effects in treating COVID-19. However, there have been contradictory results on the association between ADAM17 and the infectivity of SARS-CoV-2. One study showed higher SARS-CoV-2 viral loads in the lungs of mice upon inhibition of ADAM17 activity [17], whereas the inhibition of ADAM17 activity in cell cultures markedly reduced viral replication [21]. These findings raise some concerns and suggests that the timing of ADAM-17 inhibition may be crucial, which warrants further investigation.

A better understanding of the mechanisms associated with the expression and shedding of mACE2 could help to recognize the vast span in COVID-19 severity observed between different individuals [22,29]. We found that sACE2 correlated positively and most strongly with the ADAM-17 substrate-score, followed by renin, and IL-6. Theoretically, this could suggest a joint mechanism implicated in mACE2 shedding, dysregulated RAS signaling, and dysregulated immune regulation that is related to increased cellular ADAM-17 activity [29].

Plasma sACE2 levels were also associated (to a lesser degree) with the epithelial cell injury marker KRT19 [26], and the lung injury marker SP-D [30]. These findings indicate that plasma sACE2 levels may also reflect direct pulmonary injury and cell injury of ACE2-expressing cells, as suggested by others [26,28,31].

AREG is highly expressed in the lung and has a key involvement in many inflammatory processes but also in ARDS [32]. An impaired interferon response to COVID-19 predicts severe disease [25], and it has been proposed that at least IFN-gamma can suppress ADAM-17 activity [33]. Nevertheless, plasma sACE2 only correlated with AREG and IFN-gamma on admission. It is possible that this is associated with increased viral infection early in the disease course, whereas later in the disease course, hyperinflammation is the predominant factor driving COVID-19 pathogenesis. This can be illustrated by the relatively late spike of C-reactive protein and neutrophils, without any obvious super infection, among intubated COVID-19 patients [34]. The overall association between plasma sACE2, the ADAM-17 substrate score, renin, and inflammatory markers was stronger than the correlation between plasma sACE2 and lung injury markers, suggesting that plasma sACE2 levels are, to a greater extent, related to increased ADAM-17 activity, inflammation-induced membrane bound shedding of mACE2 and dysregulation of the RAS rather than direct lung cell injury, secondary to viral infection.

Based on age, sex, genetics, and underlying chronic diseases affecting the activity of ADAM-17 and shedding of mACE2, it is possible that the response to SARS-CoV-2 infection triggers varying cellular responses and pathological activation of signaling pathways. This could explain why some individuals are at high risk of severe COVID-19 [35]. While not directly addressed in the present study, this warrants further investigation.

Recent randomized trials were unable to demonstrate positive effects on clinical outcomes in severe COVID-19 by modulating the RAS. These studies investigated the effect of blocking ANGII and increasing ANG1-7 by the administration of synthetic ANG1-7 or an ANGII type 1 receptor–biased ligand [36]. However, clinical studies in humans on the potential benefit of selective ADAM-17 inhibition on COVID-19 are lacking.

Using data from the GTEx Portal, the fold difference between *ADAM-17* and *ACE2* gene expression was higher in the lung compared to the other tissues of interest. We therefore speculate that, upon severe acute respiratory syndrome coronavirus 2 (SARS-CoV-2) infection, there may be an increased risk of critical mACE2 deficiency emerging at a more rapid pace in the lung compared to the other tissues. However, it was shown in vitro that in alveolar epithelial cells, SARS-CoV-2 infection increases the expression of ACE2 and ADAM-17, possibly supporting these as interacting factors in the development of lung fibrosis [27].

In data from animal models on SARS-CoV-1, mACE2 does not only function as the entry receptor, but protects from acute lung injury [26,37]. This mechanism may explain why recombinant ACE2 and renin–angiotensin system blockage can protect against ALI in animal models [38,39]. Since SARS-CoV-2 cell infection can lead to ADAM-17-induced shedding of mACE2, a more pronounced reduction in the protective effects of mACE2 may follow [40] and lead to the decreased activation of anti-inflammatory, anti-fibrotic and anti-thrombotic pathways [40,41]. However, there may be differences in the age-and sex-related gene and protein expression of *ACE2* and *ADAM-17* between tissues and species, as exemplified by this and other studies. One study found an age-associated decline in lung ACE2 protein content, particularly in male mice [42]. Others showed that the gene expression of *ACE2* in nasal epithelium was lower in children compared to adults [43]. Based on the findings of the present study, we suggest that future studies should consider the eventual combined effects of *ACE2* and *ADAM-17* gene expression on mACE2 protein levels.

One strength of the present study is that data from both COVID-19 patients and non-COVID-19 patients were included. We also studied the relationship of *ACE2* and *ADAM-17* gene expression, not only *ACE2*, and therefore add important information compared to recent studies with data only on *ACE2* gene expression [43,44,45]. Furthermore, in two recent studies [44,45] also using the GTEx data, where age and sex differences in *ACE2* expression in human tissues were examined, batch effects, such as sequencing platform (Illumina HiSeq 2000 or HiSeq X) and sequencing protocol (PCR-based or PCR-free), were not considered. This may have affected the outcome of these studies. We adjusted for both sequencing platforms and protocols in our analysis, and used well-normalized gene expression, primarily designed for eQTL analysis.

Nevertheless, there are also several important aspects that could not be addressed in the present study. With regards to the MGH COVID-19 study, we could not assess if plasma sACE2 levels were related to the total amount of mACE2, level of viral load, a dissemination of SARS-CoV-2 systemically, and the infection of other organs than the lung. In addition, data on sex were not available from the openly accessible database, and others have shown that sACE2 levels are higher in men than women [46,47,48]. One study found that levels were approximately 23% higher in men than women using the same OLINK platform used in the present study (mean difference: normalized protein expression = 0.3) [48]. In addition, the role of physiological levels of sACE2 as a competitive SARS-CoV-2 agent [49] could not be addressed in this study, as viral load data were lacking. We did not directly measure cellular ADAM-17 activity; rather, a surrogate, i.e., the ADAM-17 substrate score was measured. We suggest that assessing the mean concentrations of several ADAM-17 substrates (n = 33) provides a better estimation of cellular ADAM-17 activity, than if any individual concentration was assessed. We argue that if we had only assessed the plasma concentration of one individual ADAM-17 substrate, the risk of it being affected by other factors than cellular ADAM-17 activity would be much greater. Additionally, sheddases other than ADAM-17 may shed mACE2. Nevertheless, under pro-inflammatory conditions, ADAM-17 is favored over ADAM-10, driven by increased iRhom2 activity [50].

With regards to the GTEx database, gene expression does not directly reflect activity, and the activity of ADAM-17 is also regulated, at the posttranslational level, by interaction with native inhibitors, native activators, adapter proteins, intracellular trafficking, and phosphorylation status [51]. Second, we assessed gene expression at the tissue and not at the cell level, which is a limitation because there may be major differences in the gene expression of different cell types within the tissue [52]. Additionally, information related to diseases, medications and smoking status was missing. This may have influenced the results, since diseases that increase with age, such as hypertension, type 2 diabetes mellitus and heart failure, may associate with an altered *ACE2* gene expression and turnover of mACE2. There are also indications that smoking, chronic obstructive pulmonary disease, and medications blocking the RAS may lead to the upregulation of tissue mACE2 [53,54]. Third, sample sizes were not evenly distributed between the different age groups, and more than 80% of the donors were aged between 40 to 70 (https://www.gtexportal.org/home/tissueSummaryPage, accessed on 1 May 2020). Furthermore, the ethnicity was not representative of the whole world population (84.6% were white). Finally, we did not have data on the consistency between gene expression and tissue protein content. However, others [55] have confirmed positive correlations between *ACE2* and *ADAM-17* gene expression and protein levels across 375 cell lines (r = 0.67 and r = 0.45, respectively).

## 4. Material and Methods

### 4.1. Retrieval of MGH-OLINK-COVID-19 Dataset

Data on the study population and methods have been described in detail previously [28]. We obtained data from the publicly open Massachusetts General Hospital (MGH)-OLINK-COVID-19 study from the website https://www.olink.com/mgh-covid-study/ (accessed on 16 September 2020). The study cohort encompassed acutely ill patients admitted to the emergency department in a large, urban, academic hospital in Boston (with institutional review board approval) from late winter to early spring of 2020. Included subjects were 18 years or older with a clinical concern for COVID-19 upon emergency department arrival, and with acute respiratory distress with at least one of the following: (1) tachypnea ≥22 breaths per minute; (2) oxygen saturation ≤92% on room air; (3) a requirement for supplemental oxygen; or (4) positive-pressure ventilation. A total of 384 patients were enrolled. Patients were classified as COVID-19-positive if they had tested positive for SARS-CoV-2 prior to enrollment or during hospitalization (n = 306, 80%). COVID-19-positive patients had their blood sampled on days 0, 3, and 7. All data published in the public domain were anonymized, and information on sex and ethnicity was absent. In the MGH-OLINK-COVID-19 database, patients were divided into categories according to age: 20–34, 36–49, 50–64, 65–79, and 80+ years. Patients were divided into categories according to BMI: underweight <18.5 kg/m^2^, normal weight 18.5–24.9, overweight 25.0–29.9, and obese 30.0–39.9, ≥40.0. Pre-existing comorbidities were classified as follows: pre-existing heart disease (coronary artery disease, congestive heart failure, valvular disease), pre-existing lung disease (asthma, chronic obstructive pulmonary disease, requiring home oxygen therapy, any chronic lung conditions), pre-existing kidney disease (chronic kidney disease, baseline creatinine >1.5 mg/dL, end-stage renal disease), pre-existing diabetes (pre-diabetes, insulin and non-insulin dependent diabetes), pre-existing hypertension, and pre-existing immunocompromised condition (active cancer, chemotherapy, transplant, immunosuppressant agents, asplenia). Respiratory symptoms (sore throat, congestion, productive or dry cough, shortness of breath), fever, and gastrointestinal symptoms (abdominal pain, nausea, vomiting, diarrhea) at presentation were retrieved. Patients were classified according to the World Health Organization (WHO) COVID-19 outcomes scale: 1 = death within 28 days, 2 = intubated, ventilated, 3 = non-invasive ventilation or high-flow nasal cannula, 4 = hospitalized, supplementary O_2_ required, 5 = hospitalized, no supplementary O_2_ required, and 6 = not hospitalized. For the analyses in the present study, we defined patients with scores 1–3 as those with severe illness, and scores 4–6 as those with non-severe illness, based on the WHO COVID-19 outcome scale.

The following data were retrieved: biomarker levels of ADAM-17 substrates (IL1R2, IL6R, Fractalcine, MCSFR, TNFR2, LDLR, SORT1, TNFalpha, Hb-EGF, AREG, FLT-3L, DLL1, Notch1, IGF2-R, HER4, LYPD3, SEMA4D, Syndecan1, Syndecan4, Vasorin, ALCAM, L-selectin, Desmoglein 2, EpCAM, ICAM-1, JAM-A, L1-CAM, NCAM, Nectin-4, APP, GP1ba, GPVi, ACE2), renin, inflammatory cytokines IL-1-beta, IL-6 and IFN-gamma, and lung injury markers SP-D, RAGE and KRT19 on admission (day 0), day 3 and day 7, as well as patient characteristics (age, BMI, respiratory symptoms, febrile symptoms, gastrointestinal symptoms), comorbidities and data on disease severity day 0, day 3, day 7 and 28-day outcomes according to the World Health Organization (WHO) COVID-19 outcomes scale. Information on 28-day mortality was also retrieved.

### 4.2. Laboratory Methods

The following known ADAM-17 substrates accessible from the MGH-OLINK COVID-19 study were retrieved: IL1R2, IL6R, Fractalcine, MCSFR, TNFR2, LDLR, SORT1, TNF-alpha, Hb-EGF, AREG, FLT-3L, DLL1, Notch1, IGF2-R, HER4, LYPD3, SEMA4D, Syndecan1, Syndecan4, Vasorin, ALCAM, L-selectin, Desmoglein 2, EpCAM, ICAM-1, JAM-A, L1-CAM, NCAM, Nectin-4, APP, GP1ba, GPVi, and ACE2, as well as renin, IL-1Beta, IL-6, IFN-gamma, SP-D, RAGE and KR1T19. All markers were analyzed by Olink^®^ Inflammation and Cardiometabolic panels as part of the MGH-OLINK COVID-19 study. The laboratory method was the Olink Proximity Extension Assay (PEA), which enables high-multiplex analysis of about 1400 plasma proteins. The initial step involves an immunoreaction with monoclonal or polyclonal antibodies (PEA probes). In this process, target proteins are bound in a pair-wise manner to prevent cross-reactive events. This is followed by a nucleotide extension part, where the oligonucleotides come in close proximity and hybridize, generating a unique sequence used for digital identification of the specific protein assay. Eventually, a detection and readout method (next-generation sequencing, NGS) is performed. In the quality control, internal control is integrated as well as samples for negative control and a reference plasma control, which are used and monitored. The coefficient of variation (CV) for between runs and within runs were also supervised. Data are presented as Normalized Protein eXpression (NPX) values, which is an arbitrary unit on a log2-scale (Olink Proteomics AB, Uppsala, Sweden; http://www.olink.com, accessed on 16 September 2020).

### 4.3. Estimation of ADAM-17 Activity

In an attempt to estimate cellular ADAM-17 activity, we identified the following 33 known ADAM-17 substrates from the OLINK panels: IL1R2, IL6R, Fractalcine, MCSFR, TNFR2, LDLR, SORT1, TNFalpha, Hb-EGF, AREG, FLT-3L, DLL1, Notch1, IGF2-R, HER4, LYPD3, SEMA4D, Syndecan1, Syndecan4, Vasorin, ALCAM, L-selectin, Desmoglein 2, EpCAM, ICAM-1, JAM-A, L1-CAM, NCAM, Nectin-4, APP, GP1ba, GPVi and ACE2 [56]. For COVID-19 positive individuals and controls, we calculated the Z score for each ADAM-17 substrate and thereafter calculated the mean Z score for all ADAM-17 substrates as an estimate of cellular ADAM-17 activity. We refer to this entity as the ADAM-17 substrate z-score. sACE2 was excluded from this score.

### 4.4. Retrieval of GTEx Datasets

Gene expressions of ACE2 and ADAM-17 for arteries (aorta, coronary and tibial), lung, heart (left ventricle and atrial appendage), kidney (cortex), colon (transverse and sigmoid), and the small intestine were queried via the GTEx Portal (https://gtexportal.org, accessed on 1 May 2020), presented as transcripts per million (TPM) for each gene per tissue. We filtered out genes with mean TPM across tissue <0.5 to analyze only stably expressed genes. The tibial artery (TPM of 0.4 for ACE2) was thus excluded from further analysis, leaving 9 tissues of interest included in this study. To account for batch effects in the further analysis, the processed, filtered, and normalized gene expression and covariates for 9 human tissues were downloaded from the GTEx Portal (https://www.gtexportal.org/home/datasets, accessed on 1 May 2020), along with the de-identified sample annotations (GTEx_v8_Annotations_SampleAttributesDS.txt). For each gene, expression values were normalized across samples using an inverse normal transformation, as described in the GTEx Portal. These well-normalized gene expressions were primarily used for expression quantitative trait loci (eQTL) analysis, whereas only genotyped samples were included. This process may result in a different sample size per tissue compared to the same tissue when gene-expression quantified.

### 4.5. Statistics

#### 4.5.1. MGH-OLINK-COVID-19 Dataset

To assess the association between the ADAM-17 substrate score, severe COVID-19 and 28-day mortality, the odds ratio (OR) was calculated. The coefficient was calculated from a linear mixed model using the ADAM-17 substrate score as an independent variable adjusted by comorbidities, age, and BMI categories. Subjects were treated as random effects, i.e., taking repeated measures (days 0, 3 and 7) into account. Effect size is presented as OR (95% confidence interval (CI)) per 1 standard deviation (SD) increase.

To assess the relationship between sACE2 and other biomarkers, we calculated the proportion of variance explained (R squared, also termed R2) and 95% CI from a linear mixed model taking age, BMI categories and comorbidities; subjects were treated as random effects, i.e., taking repeated measures into account.

The reported *p*-values are 2-tailed, and the level of significance was set at *p* < 0.05.

#### 4.5.2. GTEx Dataset

Using the GTEx data, we conducted correlation analysis by implementing linear regression on gene expression and age group (i.e., 20–29, 30–39, 40–49, 50–59, 60–69, 70–79 years) adjusted by sequencing platform (Illumina HiSeq 2000 or HiSeq X), sequencing protocol (PCR-based or PCR-free) and sex, if both males and females were considered.

Interaction effect $\beta_{3}$ between age groups and sex were estimated from a model as shown in Equation (1):

(1) $$Gene=\beta_{0}+\beta_{1}*Age+\beta_{2}*Sex+\beta_{3}*Age*Sex+\beta_{4}*platform+\beta_{5}*protocol$$

Interaction effect $\beta_{3}$ between ADAM-17 and sex were estimated from a model, as shown in Equation (2):

(2) $$ACE2=\beta_{0}+\beta_{1}*ADAM17+\beta_{2}*Sex+\beta_{3}*ADAM17*Sex+\beta_{4}*Age+\beta_{5}*platform+\beta_{6}*protocol$$

Statistical analyses were performed using R and the obtained relationship between the dependent variable (e.g., ACE2) and variable of interest (e.g., age) was presented as a beta coefficient (β) with standard error. The reported *p*-values are 2-tailed, and the level of significance was set at *p* < 0.05.

## 5. Conclusions

In conclusion, this study indicates that severe COVID-19 is associated with increased ADAM-17 activity, with possible implications for the risk of associated mortality. Although speculative, our findings furthermore indicate a bidirectional relationship between mACE2 shedding via increased ADAM-17 activity and dysregulated immune signaling. Furthermore, soluble ACE2 levels in COVID-19 may, to some extent, reflect dysregulated RAS-signaling and cell/lung tissue injury. In non-COVID-19 infected individuals, the fold difference between *ADAM-17* and *ACE2* gene expression is higher in the lungs compared to that of other tissues. These findings may be of importance as to why the lung is the most severely affected organ by COVID-19; however, further evaluation is needed in prospective studies.

## Data Availability

All data generated or analyzed during this study are publicly available and free from the MGH COVID-19 STUDY (https://www.olink.com/mgh-covid-study/, accessed on 16 September 2020) and the GTEx Portal (https://gtexportal.org, accessed on 1 May 2020). Supporting data are provided and attached to the manuscript submission (Supplementary Materials).

## Supplementary Materials

The following supporting information can be downloaded at: https://www.mdpi.com/article/10.3390/ijms25115911/s1.

## Abbreviations

ACE2: angiotensin-converting enzyme 2; ADAM-17: a disintegrin and metalloproteinase-17; ANG: angiotensin; ARDS: acute respiratory distress syndrome; COVID-19: coronavirus disease 2019; CV: coefficient of variation; HPA: human protein atlas; mACE2: membrane-bound angiotensin-converting enzyme 2; NGS: next-generation sequencing; NPX: normalized protein expression; NX: normalized expression; eQTL: quantitative trait loci; PEA: proximity extension assay; RAS: renin–angiotensin system; sACE2: soluble angiotensin-converting enzyme 2; SARS- CoV: severe acute respiratory syndrome coronavirus; TPM: transcripts per million; IFN: interferon.
