# Supplementary material for: ADAM-17 Activity and Its Relation to ACE2: Implications for Severe COVID-19
Source: Int J Mol Sci. 2024 May 29;25(11):5911. doi: 10.3390/ijms25115911 (PMC11172796; doi:10.3390/ijms25115911)
Supplement: Supplementary file 1 [file ijms-25-05911-s001.zip › ijms-2990467-supplementary.pdf]

Supplementary materials

ADAM-17 activity and its relation to ACE2: implications for severe COVID-19

Jiangming Sun[1], Andreas Edsfeldt[1,2,3], Joel Svensson[4], Toralph Ruge?, Isabel Goncalves[1,2], Per Swärd[5]

[1]Cardiovascular Research-Translational Studies, Department of clinical sciences Malmö, Lund University, Sweden
[2]Department of Cardiology, Skåne University Hospital, Sweden
[3] Wallenberg Center for Molecular Medicine, Lund University
[4] Department of Laboratory Medicine, Lund University, Sweden
[5] Clinical and Molecular Osteoporosis Research Unit, Departments of Orthopedics and Clinical Sciences, Skåne University Hospital, Lund University, Malmö, Sweden

Corresponding author: Per Swärd MD. PhD. Clinical and Molecular Osteoporosis Research Unit, Department of Orthopedics, Skåne University Hospital, SE - 205 02 Malmö, Sweden, Tel +4640331000; Fax+4640336200; E-mail: per.sward@skane.se

Table of Contents

Table S1	3
Table S2	4
Table S3	5
Table S4	6

Table S1. Linear relationship between ACE2 and age in males, females and both sexes using the GTEx data (https://gtexportal.org).

Tissue	b	se	t	p	n
Artery-Aorta	-0.13	0.04	-3.31	1.0×10^{-3}	387
Male	-0.11	0.05	-2.19	0.03	249
Female	-0.16	0.06	-2.60	0.01	138
Artery-Coronary	0.05	0.06	0.88	0.38	213
Male	0.12	0.07	1.67	0.10	129
Female	-0.06	0.10	-0.60	0.55	84
Colon-Sigmoid	-0.07	0.04	-1.61	0.11	318
Male	-0.09	0.05	-1.71	0.09	205
Female	-0.03	0.07	-0.41	0.69	113
Colon-Transverse	-0.16	0.04	-4.14	4.2×10^{-5}	368
Male	-0.21	0.04	-4.69	4.6×10^{-6}	232
Female	-0.07	0.07	-1.01	0.31	136
Heart-Atrial Appendage	0.02	0.05	0.45	0.66	372
Male	0.00	0.06	0.08	0.94	253
Female	0.05	0.08	0.66	0.51	119
Heart-Left Ventricle	-0.01	0.04	-0.16	0.87	386
Male	0.03	0.05	0.55	0.58	264
Female	-0.07	0.08	-0.88	0.38	122
Kidney-Cortex	0.01	0.11	0.05	0.96	73
Male	0.07	0.11	0.63	0.53	55
Female	-0.18	0.35	-0.52	0.61	18
Lung	0.00	0.04	-0.09	0.93	515
Male	-0.02	0.04	-0.39	0.69	349
Female	0.02	0.07	0.35	0.73	166
Small Intestine-Terminal Ileum	0.01	0.05	0.23	0.82	174
Male	-0.08	0.07	-1.19	0.24	111
Female	0.16	0.08	1.99	0.05	63

b: beta coefficient; se: standard error; t: t-statistic; p: p-value; n: sample size.

Table S2. Interaction effect of age group and sex in ACE2 gene expression using the GTEx data (https://gtexportal.org).

tissue	b	se	t	p	n
Artery-Aorta	-0.05	0.08	-0.64	0.52	387
Artery-Coronary	-0.18	0.12	-1.48	0.14	213
Colon-Sigmoid	0.05	0.08	0.58	0.56	318
Colon-Transverse	0.11	0.08	1.42	0.16	368
Heart-Atrial Appendage	0.03	0.10	0.35	0.73	372
Heart-Left Ventricle	-0.10	0.09	-1.16	0.25	386
Kidney-Cortex	-0.30	0.27	-1.10	0.27	73
Lung	0.04	0.08	0.52	0.61	515
Small Intestine-Terminal Ileum	0.24	0.11	2.30	0.02	174

b: beta coefficient; se: standard error; t: t-statistic; p: p-value; n: sample size.
Coefficient for interaction term was estimated from the model ACE2 ~ age-group + sex + age-group:sex + pcr + platform

Table S3. Linear relationship between ADAM-17 and age in males, females and both sexes using the GTEx data (https://gtexportal.org).

Tissue	b	se	t	p	n
Artery-Aorta	-0.01	0.04	-0.33	0.74	387
Male	0.00	0.05	0.00	1.00	249
Female	-0.03	0.07	-0.47	0.64	138
Artery-Coronary	0.06	0.06	1.12	0.27	213
Male	0.08	0.08	1.01	0.32	129
Female	0.02	0.08	0.20	0.84	84
Colon-Sigmoid	-0.23	0.04	-5.78	1.8×10^{-8}	318
Male	-0.23	0.05	-4.67	5.5×10^{-6}	205
Female	-0.22	0.06	-3.43	8.4×10^{-4}	113
Colon-Transverse	-0.13	0.04	-3.42	7.0×10^{-4}	368
Male	-0.10	0.05	-2.18	0.03	232
Female	-0.18	0.07	-2.63	9.7×10^{-3}	136
Heart-Atrial Appendage	0.02	0.05	0.50	0.62	372
Male	-0.02	0.06	-0.31	0.75	253
Female	0.11	0.08	1.40	0.17	119
Heart-Left Ventricle	-0.08	0.04	-1.80	0.07	386
Male	-0.05	0.06	-0.97	0.33	264
Female	-0.12	0.07	-1.82	0.07	122
Kidney-Cortex	0.25	0.10	2.46	0.02	73
Male	0.29	0.11	2.53	0.01	55
Female	0.08	0.28	0.29	0.78	18
Lung	0.03	0.04	0.73	0.46	515
Male	0.03	0.05	0.69	0.49	349
Female	0.02	0.06	0.31	0.75	166
Small Intestine-Terminal Ileum	-0.11	0.05	-2.06	0.04	174
Male	-0.08	0.07	-1.22	0.22	111
Female	-0.16	0.09	-1.78	0.08	63

b: beta coefficient; se: standard error; t: t-statistic; p: p-value; n: sample size.

Table S4. Interaction effect of age group and sex in ADAM-17 gene expression using the GTEx data (https://gtexportal.org).

Tissue	b	se	t	p	n
Artery-Aorta	-0.03	0.08	-0.42	0.68	387
Artery-Coronary	-0.05	0.12	-0.44	0.66	213
Colon-Sigmoid	0.00	0.08	0.00	1.00	318
Colon-Transverse	-0.09	0.08	-1.14	0.26	368
Heart-Atrial Appendage	0.13	0.10	1.25	0.21	372
Heart-Left Ventricle	-0.07	0.09	-0.73	0.47	386
Kidney-Cortex	-0.18	0.26	-0.69	0.50	73
Lung	-0.01	0.08	-0.11	0.91	515
Small Intestine-Terminal Ileum	-0.08	0.11	-0.73	0.47	174

b: beta coefficient; se: standard error; t: t-statistic; p: p-value; n: sample size.
Coefficient for interaction term was estimated from the model ADAM-17 ~ age-group + sex + age-group:sex + pcr + platform
